# Time trend of measles burden on children and adolescents in BRICS-plus countries from 1990 to 2021 and prediction to 2032

**DOI:** 10.3389/fmicb.2025.1612124

**Published:** 2025-07-24

**Authors:** Hongxia Yuan, Bingju Yan, Yiran Chong, Le Wang, Yong Jiang

**Affiliations:** ^1^Division of Infectious Diseases, The First Affiliated Hospital of Jinzhou Medical University, Jinzhou, China; ^2^Collaborative Innovation Center for Prevention and Control of Zoonoses of Jinzhou Medical University, Jinzhou, China; ^3^Department of Cardiology, The First Affiliated Hospital of Jinzhou Medical University, Jinzhou, China; ^4^Division of Hyperbaric Oxygen, The First Affiliated Hospital of Jinzhou Medical University, Jinzhou, China; ^5^Modern Industrial School of Health Management, Jinzhou Medical University, Jinzhou, China

**Keywords:** measles, BRICS-plus, joinpoint, age-period-cohort model, Bayesian age-period-cohort, ARIMA

## Abstract

**Background:**

Measles remains a major disease burden on children and adolescents in BRICS-plus countries (Brazil, Russia, India, China, South Africa, and five others) despite vaccine efficacy. This study aims to clarify the temporal trend of measles burden and forecast the trend in 2032.

**Methods:**

Data from the Global Burden of Disease (GBD) 2021 were utilized to analyze the prevalence, incidence, mortality, and disability-adjusted life years (DALYs) of measles in BRICS-plus countries. In addition, the association between the social development index (SDI) and measles-related indicators of children and adolescents in BRICS-plus countries was analyzed. Joinpoint regression was performed to identify temporal trends, while the age-period-cohort model was used to assess demographic effects. The Bayesian Age-Period-Cohort (BAPC) and Autoregressive Integrated Moving Average (ARIMA) models were utilized to project indicators to 2032.

**Results:**

From 1990 to 2021, the global prevalence of measles dropped by 92% (with an average annual decline of 6.80%), and the average annual decline rates for incidence, mortality, and DALYs were 6.80, 8.02, and 8.02%, respectively. Saudi Arabia had a 100% reduction in prevalence (with an average annual decrease of 15.20%), Ethiopia had the highest DALYs (124542.02), and Russia had the lowest DALYs (1.74). SDI was negatively linked to the measles prevalence (*R* = −0.703, *p* < 0.001), and an increase in SDI significantly reduced the burden of measles. The prevalence of measles was highest among children under 5 years old and slightly higher in males than in females. Joinpoint analysis indicated that the global burden of measles declined, but its mortality in China sharply increased from 2019 to 2021 (APC = 191.88). The BAPC model predicted that by 2032, the global burden of measles will continue to decline, India will still have the highest prevalence (130.96), Russia may have no new cases, and Brazil and South Africa will have controllable local risks. ARIMA models showed similar trends.

**Conclusion:**

The declining burden of measles in BRICS-plus countries is correlated with SDI improvement, but low-income countries such as Ethiopia still face a high burden of measles. Children under 5 years and regions with low vaccination rates require prioritized interventions. The burden of measles will continue to decrease in the next decade, and increasing vaccination coverage in high-burden countries will help achieve the goal of measles elimination.

## 1 Introduction

Measles is an acute respiratory infectious disease resulting from the measles virus. Its basic reproduction number (*R*_0_) is as high as 12–18, indicating extremely high infectivity (Moss, 2017; Guerra et al., 2017). From 2000 to 2018, the global incidence of measles greatly decreased from 145 cases per million people to 49 cases, with a reduction of 66% (Gastañaduy et al., 2021). However, in 2018, global measles cases sharply increased by 167% compared to 2016 (Patel et al., 2019). In particular, the number of cases in Africa surged to nearly 290,000. According to the latest estimates by the World Health Organization (WHO) and the United States Centers for Disease Control and Prevention, there were approximately 10.3 million cases of measles worldwide in 2023, a 20% increase compared to 2022 (Measles).^1^ The high infectivity makes measles a chief cause of death among children globally, especially in areas with low vaccination rates (Lazar et al., 2019; Do et al., 2021; Stein-Zamir et al., 2024). In 2018, approximately 350,000 measles cases were reported globally, resulting in about 142,000 deaths, mostly in children under 5 years old (Misin et al., 2020). The Corona Virus Disease 2019 (COVID-19) pandemic severely disrupted global vaccination programs, leaving millions of children unvaccinated against measles (Do et al., 2022), ultimately leading to local resurgences of measles (Tariq et al., 2022; George et al., 2024). Although measles is a vaccine-preventable disease, it remains a significant cause of death among children and adolescents in low- and middle-income countries (Wang et al., 2021; Portnoy et al., 2019). Therefore, measles remains a serious public health issue worldwide.

The BRICS has been established since 2009, and its initial members include Brazil, Russia, India, and China. In 2011, South Africa officially joined the organization. By January 1, 2024, Saudi Arabia, Egypt, the United Arab Emirates, Iran, and Ethiopia have also become full members of the BRICS, marking the expansion of the BRICS to BRICS-plus (Cheng et al., 2025; Wang et al., 2025), representing over half of the global population. They have a relatively high proportion of children and adolescents and face challenges in controlling measles, especially in areas with insufficient vaccination coverage (Yousif et al., 2022; Costa et al., 2020; Panda et al., 2020). Brazil reported over 10,000 cases of measles between 2018 and 2019, mainly among unvaccinated people (Costa et al., 2020). Russia and South Africa exhibited a relatively low incidence of measles but still experienced local outbreaks (Yousif et al., 2022; Muscat et al., 2024). India reported hundreds of thousands of cases each year. In 2017, 2.9 million children did not receive the first dose of measles-containing vaccine (MCV) on time, and the vaccination rate in 2018 was only 86%, far below the expected 95% (Pustake et al., 2022). From October 2021 to September 2022, 172 measles outbreaks were reported, with a total of 12,589 cases (Kumar et al., 2023). The incidence of measles in China dropped to 0.06 per 100,000 in 2023, no deaths were reported for many consecutive years, and measles is being eliminated in China (Ma et al., 2019; Wang H. et al., 2023; Durrheim et al., 2023). However, local outbreaks remain (Zhang et al., 2020; Li et al., 2019). Due to vaccine hesitancy, Saudi Arabia, the United Arab Emirates, and Ethiopia are all threatened by measles to varying degrees (Al-Abdullah, 2018; Alamer et al., 2022; Barqawi et al., 2024; Shimelis et al., 2024). Reports confirm that Egypt and Iran eliminated measles between 2019 and 2022, but there have been no authoritative reports in recent years. WHO data show that the Middle East remains one of the hotspots for measles outbreaks. The measles epidemic in BRICS-plus countries has not been effectively controlled.

There is currently a lack of comprehensive research on the burden and development trend of measles in BRICS-plus countries. Therefore, this paper aims to analyze the temporal changes in measles burden among children and adolescents in BRICS-plus countries from 1990 to 2021, predict the disease trends to 2032, and strive to provide a scientific basis for decision-makers and public health workers to increase vaccination rates, reduce measles burden, and eradicate measles.

## 2 Materials and methods

### 2.1 Data source and disease definition

The 2021 Global Burden of Disease (GBD) report produced by the Institute of Health Indicators and Evaluation (IHME) provides the burden data of 371 diseases and injuries in 204 countries and regions, including the prevalence, incidence, mortality, and risk factors (GBD 2021 Demographics Collaborators, 2024; GBD 2021 Risk Factors Collaborators, 2024; GBD 2021 Causes of Death Collaborators, 2024; GBD 2021 Diseases and Injuries Collaborators, 2024). All data were available to the public free of charge through the website,^2^ including data available in previous reports, statistical modeling, and methodological information. According to GBD 2021, measles is highly contagious and mainly spreads through respiratory droplets. The typical clinical manifestations of measles include fever, cough, runny nose, conjunctivitis, oral mucosal spots, and systemic maculopapules. According to the tenth revision of the International Classification of Diseases, the code for measles is B05.

The GBD 2021 query tool was utilized to collect data on the prevalence, incidence, mortality, and disability-adjusted life years (DALYs) from 1990 to 2021 in the global measles population and among children and adolescents in BRICS-plus countries. Given the data variability, the final estimate represents the average result of 500 calculations, and the boundary of uncertainty intervals was defined by the 2.5th and 97.5th percentiles, resulting in 95% uncertainty intervals (UIs) (GBD 2021 Diseases and Injuries Collaborators, 2024). The detailed methodology and modeling process of GBD 2021 have been recorded in other related publications (GBD 2021 Causes of Death Collaborators, 2024; GBD 2021 Diseases and Injuries Collaborators, 2024). The data set used was anonymous and open to the public free of charge.

### 2.2 Socio-demographic index

Socio-demographic index (SDI) is a composite marker of lag-distributed income per capita, average years of education, and fertility rates among females under 25 years (GBD 2021 Diseases and Injuries Collaborators, 2024). It ranged from 0 to 1, with 0 indicating the lowest development level and 1 indicating the highest level. According to the SDI value of GBD 2021, countries were divided into five SDI quintiles: low SDI, low-middle SDI, middle-SDI, high-middle SDI, and high-SDI regions.

### 2.3 DALYs

DALYs is a comprehensive index to quantify the impact of diseases, injuries, and risk factors on health. It comprehensively evaluates the disease burden from disability and mortality, including years of life lost (YLLs) and years lived with disability (YLDs). DALYs are widely used to evaluate the disease burden.

### 2.4 Ethics approval

The data used were derived from the GBD study, which has been reviewed by the Institutional Review Board of the University of Washington and is available for public inquiry. The analysis of the GBD study followed the principles of accuracy and transparency and the guidelines for health estimation reports (GBD 2021 Diseases and Injuries Collaborators, 2024).

### 2.5 Statistical analysis

#### 2.5.1 Estimated annual percentage changes and percentage change

From 1990 to 2021, estimated annual percentage changes (EAPCs) were calculated to reflect the fluctuating trend of measles burden among children and adolescents. The trends can be identified in a specific period using EAPCs and their 95% confidence intervals (CIs) (Cen et al., 2024). When the upper limit of EAPC (95% CI) was lower than zero, it showed a statistically significant downward trend. On the contrary, when the lower limit of EAPC (95% CI) exceeded zero, it implied a statistically significant upward trend. If EAPC (95% CI) contained zero, it indicated no statistical significance. In addition, percentage changes were utilized to indicate the changes in prevalence, incidence, mortality, and DALYs in 2021 compared with 1990.

#### 2.5.2 Joinpoint regression program

Joinpoint regression analysis can be used to estimate disease trends using the least squares method, effectively avoiding the subjectivity of traditional linear trend analysis. This method aims to identify key turning points in disease burden trends and calculate annual percentage changes for each stage (Zhang et al., 2022), which is widely used in epidemiological research to estimate the temporal trends of disease prevalence or mortality (Wang and Miao, 2024). It can effectively identify and quantitatively describe the significant change in measles prevalence in the global and BRICS-plus countries. Through this model, the annual percentage change (APC) and its 95% CI were calculated to divide the fashion trends into different periods. To comprehensively appraise the trends, the average annual percentage change (AAPC) was calculated, which covered the comprehensive trend data from 1990 to 2021. APC or AAPC was compared with zero to determine whether the fluctuation trends in different regions were statistically significant. *p* < 0.05 inferred statistical significance.

### 2.6 Age-period-cohort modeling analysis

Due to the linear relationship between age, period, and cohort, it is difficult to estimate the unique effect set for each age, period, and cohort. Therefore, the age-period-cohort model is used to analyze the temporal trends of different age groups, periods, and birth cohorts (Zhang et al., 2022), aiming to study the temporal trend of incidence or mortality with age, period, and cohort (Fan et al., 2023). The net drift reflects the percentage change in the population in 1 year, while the local drift shows the APC in each age group. The longitudinal age curve shows the specific age rate fitted in the reference cohort, and the periodic deviation has been adjusted. Periodic relative risk (RR) was adjusted by age and nonlinear cohort effect in each period compared to the reference period. Cohort RR was adjusted by age and the nonlinear periodic effect in each cohort compared to the reference cohort. RR >1 showed that this factor increased the risk of measles; RR <1 showed that this factor reduced the risk of measles. To solve the identification problem caused by the linear relationship between age, period, and cohort, the internal estimator method associated with the age-period-cohort model was employed to overcome the unpredictability of model parameters.

### 2.7 Bayesian age-period-cohort

The Bayesian age-period-cohort (BAPC) model considers the effects of age, period, and birth cohort on disease outcomes. It uses a second-order random walk (RW 2) model to combine prior information about unknown parameters with sample data to analyze and predict the effects of age, period, and cohort on a given event (such as mortality and disease incidence) in the population (Ji et al., 2023). This model has higher accuracy in predicting the disease burden. Therefore, R-package BAPC and integrated nested Laplace approximation were leveraged to predict the burden of measles mortality and DALYs in the initial BRICS-plus countries from 2022 to 2032.

### 2.8 ARIMA

Autoregressive Integrated Moving Average (ARIMA) model is a commonly used time series analysis method. We used this model to predict the prevalence, incidence, mortality, and DALYs of measles among children and adolescents in the BRICS-plus countries from 2022 to 2032. The model can effectively capture the trend and seasonal change characteristics in time series data by integrating three major elements: autoregression (AR), difference (I), and moving average (MA). The auto.arima function was used to select the optimal model based on the Akaike information criterion, and the Ljung–Box test was used to verify whether the residual sequence was white noise.

Joinpoint and age-period-cohort analyses reveal trend characteristics, while the BAPC model clarifies the underlying drivers behind the trends. ARIMA models analyze time series data to eliminate trends and seasonal effects, revealing underlying long-term patterns while controlling for confounding factors.

In this study, the prevalence, incidence, mortality, and DALYs rates were all expressed as the predicted values per 100,000 population with a 95% UI. All analyses were performed in R software and Joinpoint Regression Program 5.3.0 (National Institutes of Health, n.d.). *p <* 0.05 (two-sided) inferred statistical significance.

## 3 Results

### 3.1 Trends in measles burden in BRICS-plus countries and globally, 1990–2021

**Figure 1:**
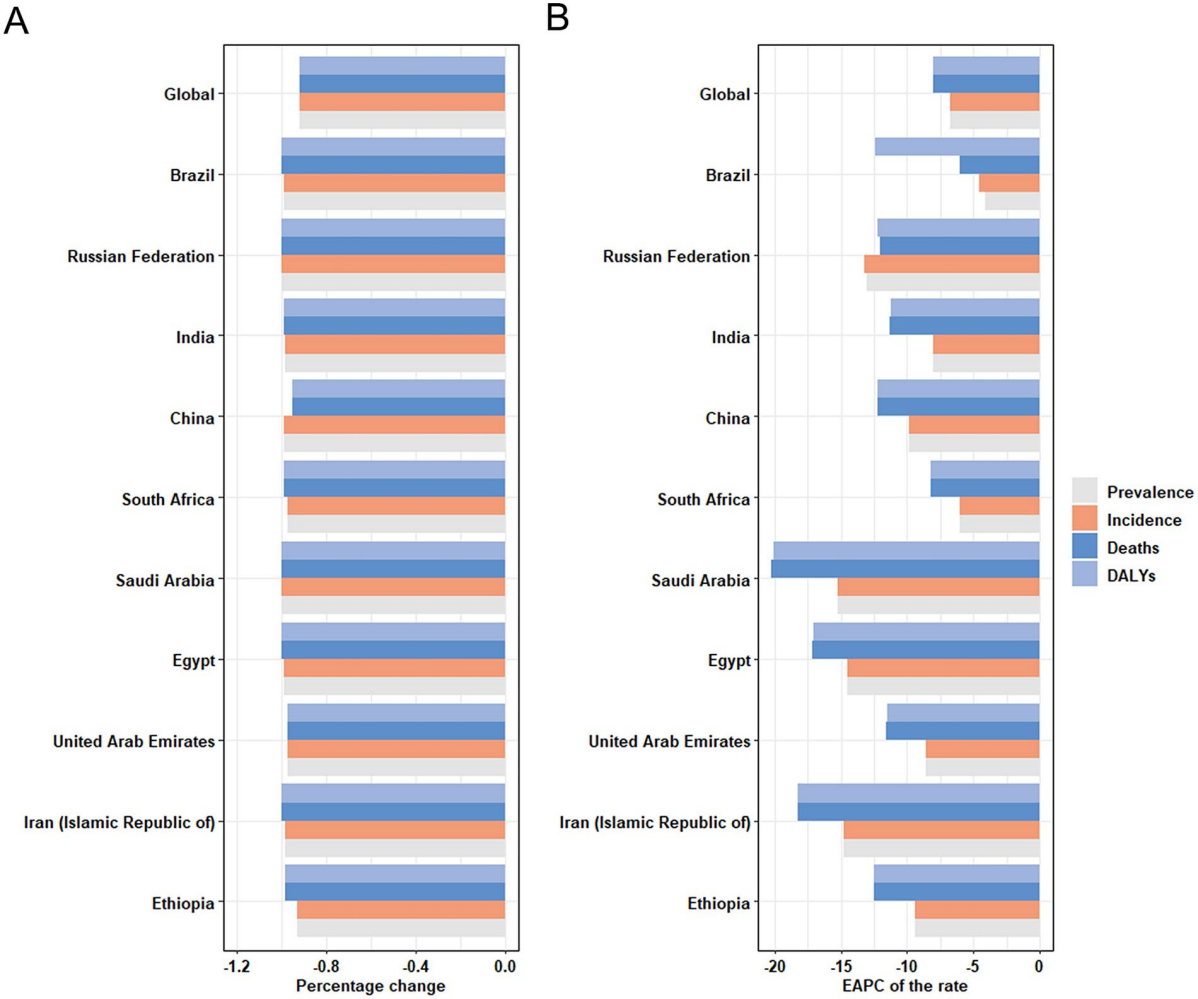
Temporal trend of disease burden of children and adolescents in global and BRICS-plus countries. **(A)** Percentage change in cases of prevalence, incidence, mortality, and DALYs in 1990 and 2021. **(B)** The EAPC of prevalence, incidence, mortality, and DALYs rates from 1990 to 2021. DALYs, disability-adjusted life years; EAPC, estimated annual percentage change.

**Figure 2:**
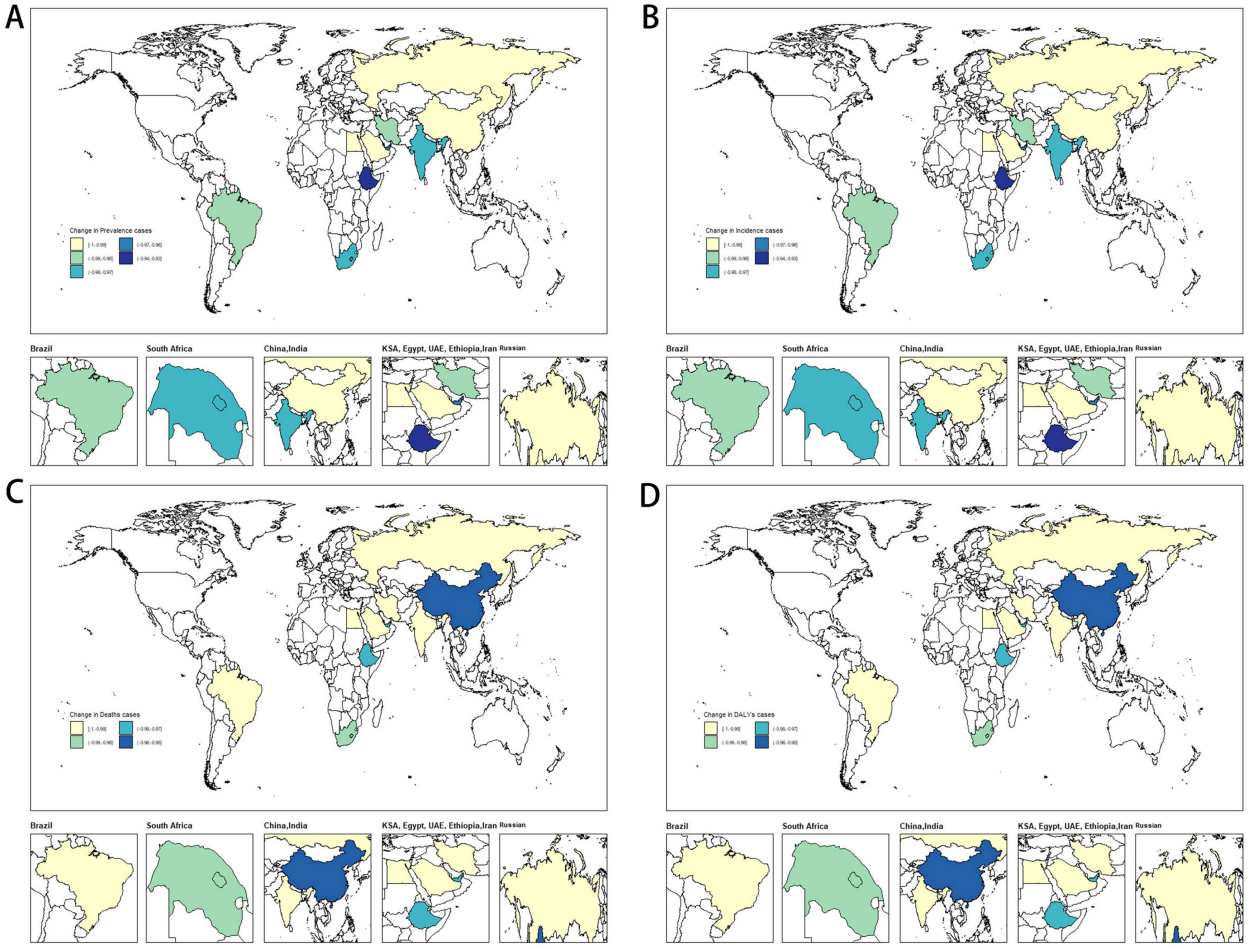
Temporal trend of disease burden of children and adolescents in BRICS-plus countries. **(A)** Percentage change in prevalent cases. **(B)** Percentage change in incident cases. **(C)** Percentage change in death cases. **(D)** Percentage change in DALYs cases. DALYs, disability-adjusted life years.

The changes in measles-related burden to children and adolescents per 100,000 people in BRICS-plus countries and over the world (1990–2021) are shown in Table 1; Supplementary Table 1 and Figures 1, 2. Between 1990 and 2021, the global annual number of measles declined from 1701026.49 (95% UI: 600598.31 to 3702216.49) to 130392.93 (95% UI: 114767.08 to 147285.75), representing a reduction of 92%, with an average annual decrease of 6.80% (95% CI: −7.74 to −5.85). Moreover, from 1990 to 2021, the incidence, mortality, and DALYs of measles among children and adolescents were all decreased, and the EAPC was −6.80 (95% CI: −7.74 to −5.85), −8.02 (95% CI: −8.58 to −7.46), and −8.02 (95% CI: −8.58 to −7.46), respectively. The overall measles burden in BRICS-plus countries showed a significant downward trend. The prevalence, incidence, mortality, and DALYs of measles were substantially reduced in Saudi Arabia. For instance, the prevalent cases decreased from 8320.57 (95% UI: 2961.13 to 18219.85) to 33.21 (95% UI: 7.34 to 68.26), representing a reduction of 100%, with an average annual decrease of 15.20% (95% CI: −18.73 to −11.53), followed by Iran and Egypt (Supplementary Table 1 and Figures 1, 2). Brazil had a smaller decline from 1607.74 (95% UI: 1594.94 to 1619.91) to 19.05 (95% UI: 17.57 to 20.51) (Table 1; Figures 1, 2). In addition, Brazil had no significant changes in incidence and mortality, with EAPCs of −4.55 (95% CI: −16.5 to 9.13) and −5.99 (95% CI: −11.7 to 0.09), respectively.

**Table 1:** Trends in the burden of measles between 1990 and 2021 across the BRICS.

Location	Num_1990 (95% UI)	Num_2021 (95% UI)	Percentage_change (100%)	Rate_1990 per 100,000 (95% UI)	Rate_2021 per 100,000 (95% UI)	EAPC (95% CI)
Prevalence						
Global	1701026.49 (600598.31–3702216.49)	130392.93 (114767.08–147285.75)	−0.92 (−0.81 to −0.96)	7531.42 (2659.19–16391.83)	494.69 (435.41–558.78)	−6.8 (−7.74 to −5.85)
Brazil	1607.74 (1594.94–1619.91)	19.05 (17.57–20.51)	−0.99 (−0.99 to −0.99)	239.27 (237.37–241.08)	2.98 (2.75–3.21)	−4.09 (−13.46 to 6.29)
Russian Federation	493.54 (486.47–500.79)	0.02 (0–0.07)	−1 (−1 to −1)	109.23 (107.66–110.83)	0.01 (0–0.02)	−13.01 (−18.4 to −7.27)
India	597505.81 (205169.4–1295779.55)	12431.4 (8325.82–16471.2)	−0.98 (−0.96 to −0.99)	14558.53 (4999.06–31572.33)	248.34 (166.33–329.05)	−8.06 (−10.16 to −5.9)
China	2289.96 (2275.22–2305.94)	18.97 (17.44–20.42)	−0.99 (−0.99 to −0.99)	51.45 (51.12–51.81)	0.57 (0.52–0.61)	−9.85 (−13.07 to −6.5)
South Africa	20345.07 (7228.8–44991.81)	565.76 (374.86–794.02)	−0.97 (−0.95 to −0.98)	11545.46 (4102.21–25532.04)	284.05 (188.2–398.64)	−6.05 (−8.62 to −3.41)
Incidence						
Global	62087466.81 (21921838.14–135130901.73)	4759421.16 (4189082.17–5376013.35)	−0.92 (−0.81 to −0.96)	274896.67 (97060.5–598301.67)	18056.56 (15892.77–20395.82)	−6.8 (−7.74 to −5.85)
Brazil	58682.64 (58215.15–59126.74)	695.34 (641.4–748.54)	−0.99 (−0.99 to −0.99)	8733.5 (8663.92–8799.59)	108.79 (100.35–117.11)	−4.55 (−16.5 to 9.13)
Russian Federation	18014.09 (17756.01–18278.68)	0.83 (0–2.55)	−1 (−1 to −1)	3986.74 (3929.62–4045.3)	0.25 (0–0.75)	−13.2 (−18.69 to −7.34)
India	21808962.03 (7488683.15–47295953.57)	453746.1 (303892.43–601198.72)	−0.98 (−0.96 to −0.99)	531386.41 (182465.56–1152389.87)	9064.55 (6070.9–12010.23)	−8.06 (−10.16 to −5.9)
China	83583.39 (83045.52–84166.68)	692.45 (636.52–745.15)	−0.99 (−0.99 to −0.99)	1878.1 (1866.02–1891.21)	20.71 (19.04–22.29)	−9.85 (−13.07 to −6.5)
South Africa	742594.96 (263851.24–1642201.03)	20650.39 (13682.36–28981.77)	−0.97 (−0.95 to −0.98)	421409.18 (149730.8–931919.32)	10367.7 (6869.34–14550.54)	−6.05 (−8.62 to −3.41)
Mortality						
Global	670151.3 (249955.15–1345889.86)	55401.2 (31949.91–85916.54)	−0.92 (−0.87 to −0.94)	2967.14 (1106.69–5959.02)	210.18 (121.21–325.95)	−8.02 (−8.58 to −7.46)
Brazil	276.83 (216.69–333.2)	0.37 (0.13–0.77)	−1 (−1 to −1)	41.2 (32.25–49.59)	0.06 (0.02–0.12)	−5.99 (−11.7 to 0.09)
Russian Federation	9.05 (5.97–13.56)	0.02 (0–0.05)	−1 (−1 to −1)	2 (1.32–3)	0.01 (0–0.01)	−12.05 (−14.97 to −9.04)
India	160229.55 (58662.92–317595.15)	1106.66 (532.65–2053.65)	−0.99 (−0.99 to −0.99)	3904.07 (1429.35–7738.37)	22.11 (10.64–41.03)	−11.29 (−13.33 to −9.2)
China	291.35 (191.19–408.59)	13.34 (8.09–20.88)	−0.95 (−0.96 to −0.95)	6.55 (4.3–9.18)	0.4 (0.24–0.62)	−12.23 (−14.73 to −9.66)
South Africa	2874.36 (999.4–6210.42)	38.46 (18.66–76.19)	−0.99 (−0.98 to −0.99)	1631.15 (567.14–3524.3)	19.31 (9.37–38.25)	−8.25 (−10.8 to −5.62)
DALYs						
Global	58581088.94 (21863110.63–117684915.66)	4839654.44 (2792705.27–7500361.47)	−0.92 (−0.87 to −0.94)	259371.93 (96800.48–521058.32)	18360.95 (10595.12–28455.29)	−8.02 (−8.58 to −7.46)
Brazil	24427.27 (19172.82–29377.2)	33.67 (12.58–69.32)	−1 (−1 to −1)	3635.41 (2853.41–4372.09)	5.27 (1.97–10.84)	−12.4 (−21.85 to −1.82)
Russian Federation	828.04 (559.07–1218.13)	1.74 (0.14–4.41)	−1 (−1 to −1)	183.25 (123.73–269.59)	0.51 (0.04–1.31)	−12.22 (−15.26 to −9.07)
India	13987643.96 (5127269.6–27707806.02)	97011.63 (46424.37–179433.74)	−0.99 (−0.99 to −0.99)	340816.03 (124928.52–675114.73)	1938.01 (927.43–3584.57)	−11.27 (−13.31 to −9.19)
China	25541.6 (16818.05–35827.67)	1158.28 (701.83–1814.5)	−0.95 (−0.96 to −0.95)	573.91 (377.9–805.04)	34.65 (20.99–54.28)	−12.19 (−14.69 to −9.61)
South Africa	252020.79 (87776.97–543866)	3339.01 (1616.75–6574.21)	−0.99 (−0.98 to −0.99)	143017.23 (49811.84–308634.1)	1676.38 (811.7–3300.64)	−8.23 (−10.79 to −5.6)

**Figure 3:**
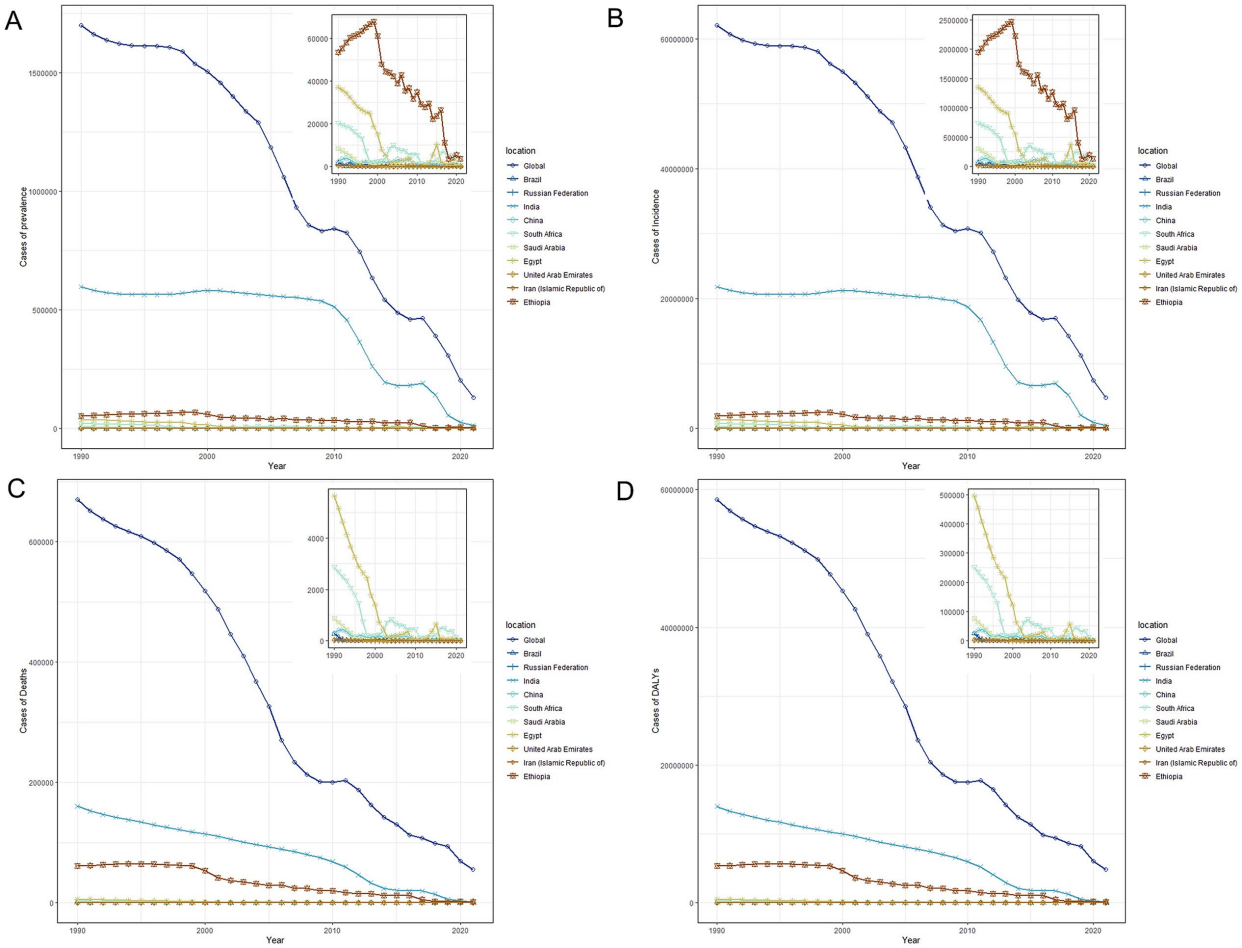
The cases of prevalence, incidence, deaths, and DALYs from 1990 to 2021. **(A)** Prevalence cases. **(B)** Incidence cases. **(C)** Mortality cases. **(D)** DALYs cases. DALYs, disability-adjusted life years.

In 2021, Ethiopia had the highest measles-associated DALYs at 124542.02 (95% UI: 64010.84 to 219173.95). In contrast, the Russian Federation reported a minimal count at 1.74 (95% UI: 0.14 to 4.41). Ethiopia also had the highest DALYs rate per 100,000 population at 21773.84 (95% UI: 11191.1 to 38318.47), while the Russian Federation presented the lowest rate per 100,000 population at 0.51 (95% UI: 0.04 to 1.31). These results showed that the burden of measles on children and adolescents in BRICS-plus countries continued to decrease (Table 1; Supplementary Figure S1; Figures 1, 2). The trends of the prevalence, incidence, mortality, and DALYs of measles in different regions over time are displayed in Figure 3. The prevalence, incidence, mortality, and DALYs of measles in the global scope and BRICS-plus countries gradually decreased. The downward trend was particularly significant in India and remained relatively stable in other countries.

### 3.2 Link between the measles burden and SDI

**Figure 4:**
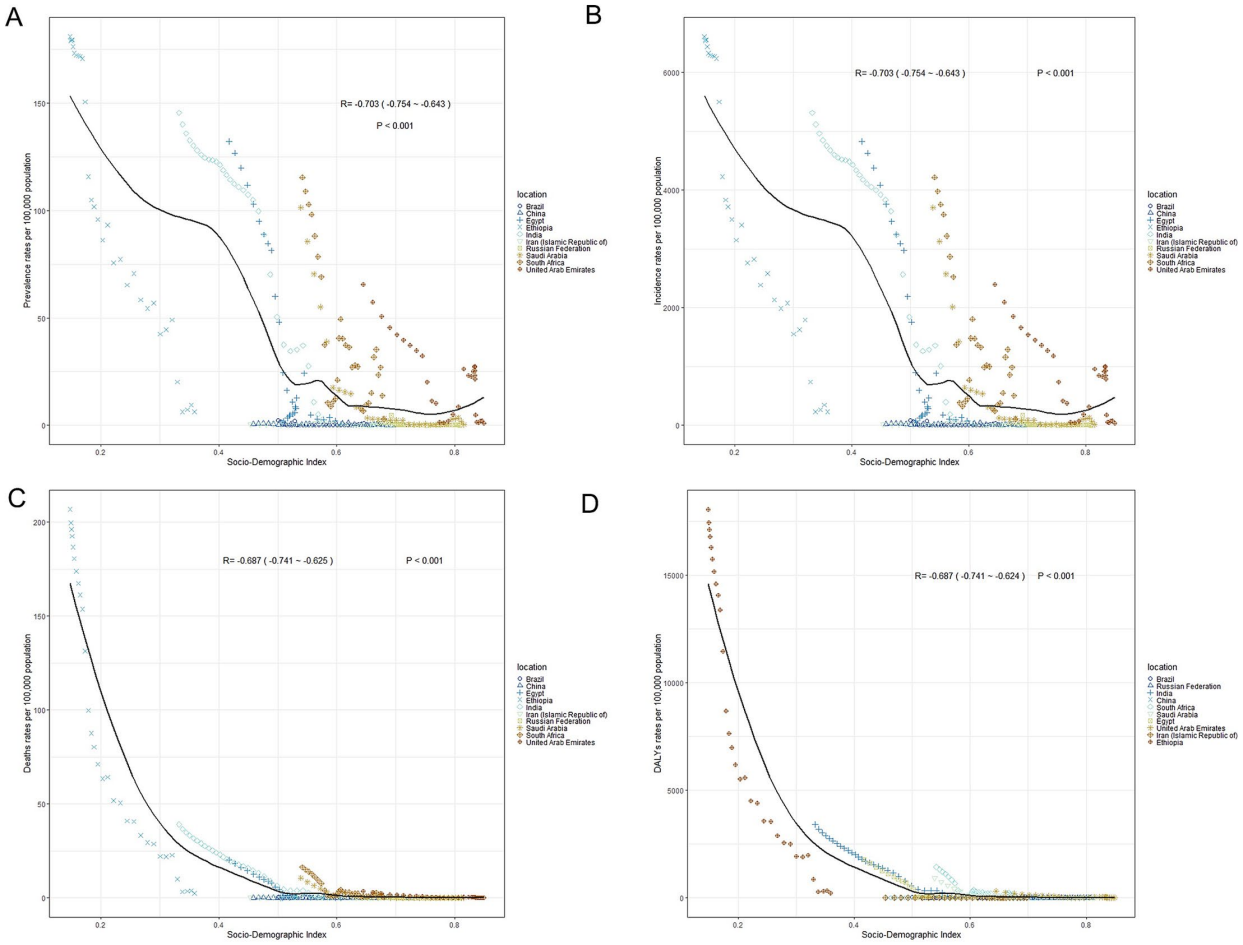
The associations between the SDI and burden of measles (per 100,000 population) in children and adolescents in BRICS-plus countries. **(A)** Prevalence rates. **(B)** Incidence rates. **(C)** Mortality rates. **(D)** DALYs rates. SDI, socio-demographic index; DALYs, disability-adjusted life years.

Significant regional differences were observed in the link between SDI and measles burden in BRICS-plus countries. In 2021, as the SDI level improved in 204 countries and regions worldwide, the burden of measles among children and adolescents in BRICS-plus countries decreased significantly, indicating a significant negative correlation between SDI and measles. From 1990 to 2021, the prevalence of measles among children and adolescents in BRICS-plus countries showed a downward trend with the increase of SDI (*R* = −0.703, *p <* 0.001). The downward trend was most apparent when SDI was between 0.00 and 0.50; countries with high-middle SDI (e.g., Russia and China) and middle SDI (e.g., Brazil and Iran) showed lower-than-expected measles prevalence (Figure 4A). Moreover, from 1990 to 2021, the incidence (*R* = −0.703, *p <* 0.001), mortality (*R* = −0.687, *p <* 0.001), and DALYs of measles (*R* = −0.687, *p <* 0.001) were negatively correlated with SDI (Figures 4B–D).

### 3.3 Age and sex patterns

**Figure 5:**
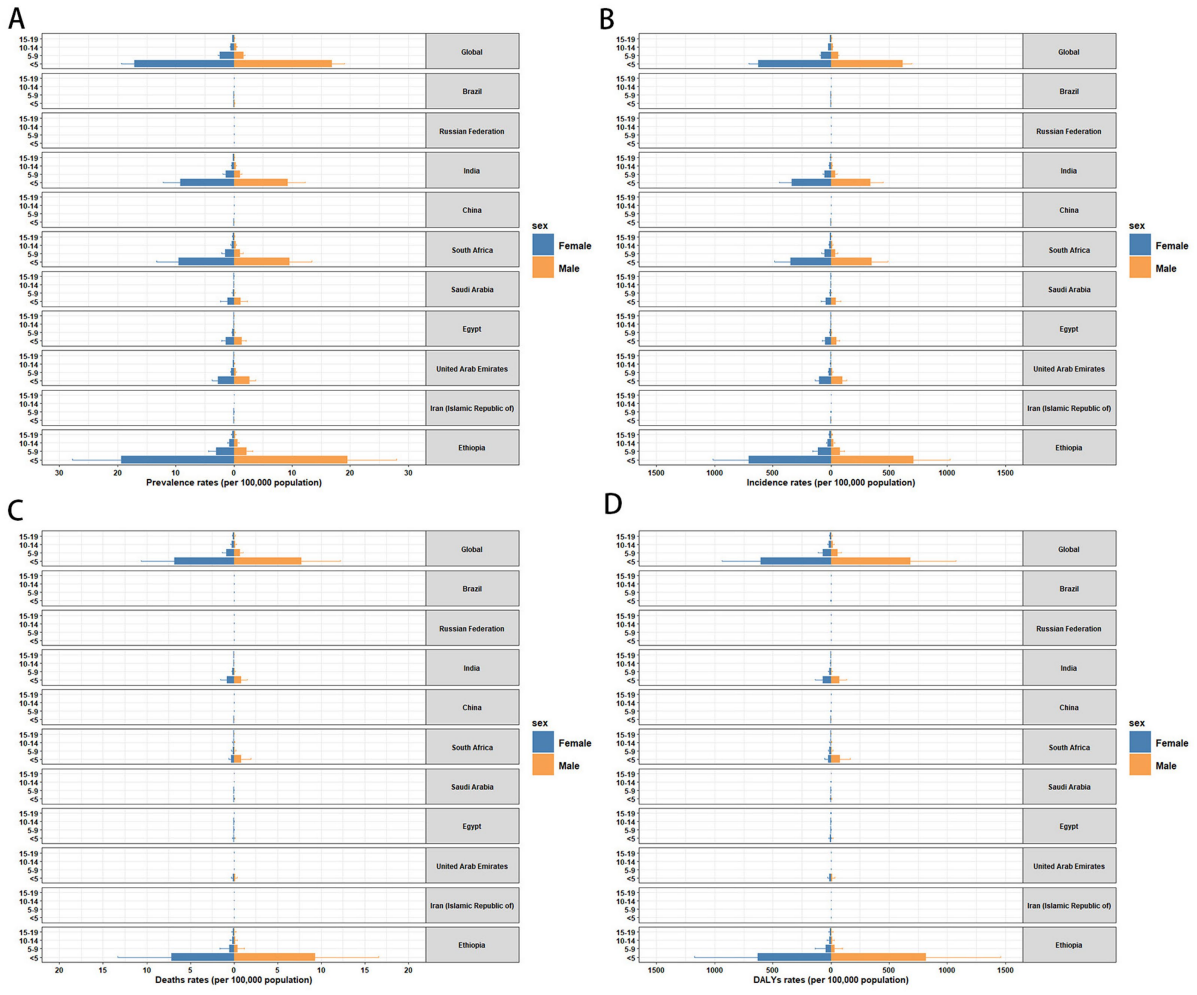
Sex- and age-structured analysis of measles burden in 2021. **(A)** Prevalence rates. **(B)** Incidence rates. **(C)** Mortality rates. **(D)** DALYs rates. DALYs, disability-adjusted life years.

Measles prevalence was higher globally in the under-five age group, with females (17.09 per 100,000) having a slightly higher prevalence than males (16.88 per 100,000). In BRICS-plus countries, the prevalence of measles was highest among children under 5 years, especially in Ethiopia (19.52 per 100,000 for males and 19.34 per 100,000 for females), followed by South Africa and India. Russia, on the other hand, showed the lowest prevalence. In addition, among children and adolescents in BRICS-plus countries, the prevalence rate of measles decreased with age (Figure 5A and Supplementary Table 2). The incidence rate of measles was higher in the under-five age group globally and in BRICS-plus countries. According to the data in 2021, Ethiopia showed the most new cases of measles, with males (712.52 per 100,000) slightly higher than females (705.91 per 100,000) (Figure 5B and Supplementary Table 2). The mortality rate of measles in the under-five age group was the highest in Ethiopia, followed by South Africa and India. However, the overall measles mortality rate was low globally and in BRICS-plus countries (Figure 5C and Supplementary Table 2). The analysis of DALYs rate showed a similar trend to the prevalence. DALYs rate gradually decreased with age. Ethiopia showed the largest difference in DALYs rate between sexes among children under 5 years (females 630.01 per 100,000 vs. males 820.53 per 100,000), followed by the age group of 5–9 years old (females 45.74 per 100,000 vs. males 33.97 per 100,000) and the age group of 10–14 years old (females 13.07 per 100,000 vs. males 11.88 per 100,000) (Figure 5D and Supplementary Table 2).

### 3.4 Temporal joinpoint analysis

**Figure 6:**
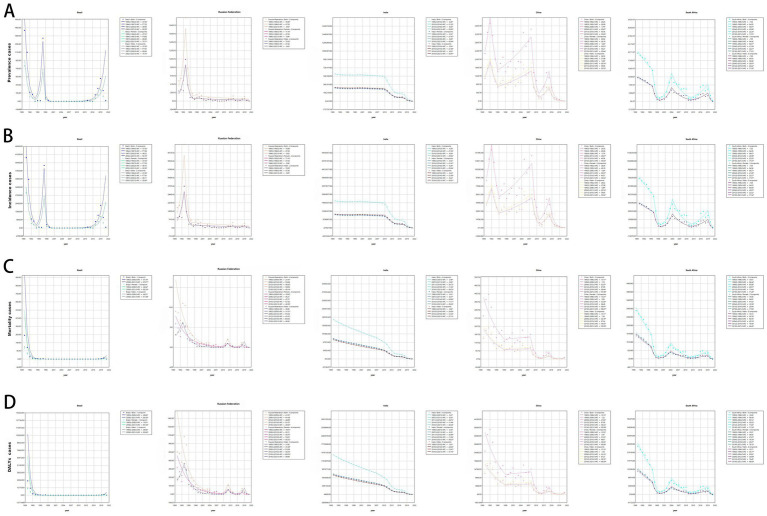
Joinpoint regression analysis of measles burden among children and adolescents in the BRICS countries. **(A)** Prevalence cases. **(B)** Incidence cases. **(C)** Mortality cases. **(D)** DALYs cases. DALYs, disability-adjusted life years.

Joinpoint regression analysis showed a downward trend in the global prevalence of measles in children and adolescents from 1990 to 2021 (AAPC = −7.94; 95% CI: −8.58 to −7.3; *p* = 0.001). However, the change in the prevalence among female patients between 2008 and 2011 was not significant (APC = −1.68; 95% CI: −12.25 to 10.16; *p* = 0.758) (Supplementary Table 3). Incidence and prevalence showed similar trends. Notably, changes in mortality and DALYs were not significant between 2008 and 2011 for either male or female patients, while other indicators demonstrated consistent trends with prevalence and incidence (Supplementary Table 3). Figure 6; Supplementary Figure S1 present joinpoint regression analyses of the prevalence, incidence, mortality, and DALYs of measles among children and adolescents in BRICS-plus countries from 1990 to 2021. The measles burden on children and adolescents in BRICS-plus countries showed a decreasing trend. However, the burden in China, South Africa, the United Arab Emirates, and Iran fluctuated significantly over time while relatively stable in other countries. With a large population base, India showed a significant downward trend in overall disease and measles burden at all stages. Despite the overall downward trend in China, the mortality (APC = 191.88; 95% CI: 4.47 to 715.47; *p* = 0.042) and DALYs of measles (APC = 183.99; 95% CI: 0.28 to 704.23; *p* = 0.049) were increased in children and adolescents between 2019 and 2021 (Figure 6; Supplementary Figure S1; Supplementary Tables 4–13).

### 3.5 Temporal trend of measles burden in different age groups

**Figure 7:**
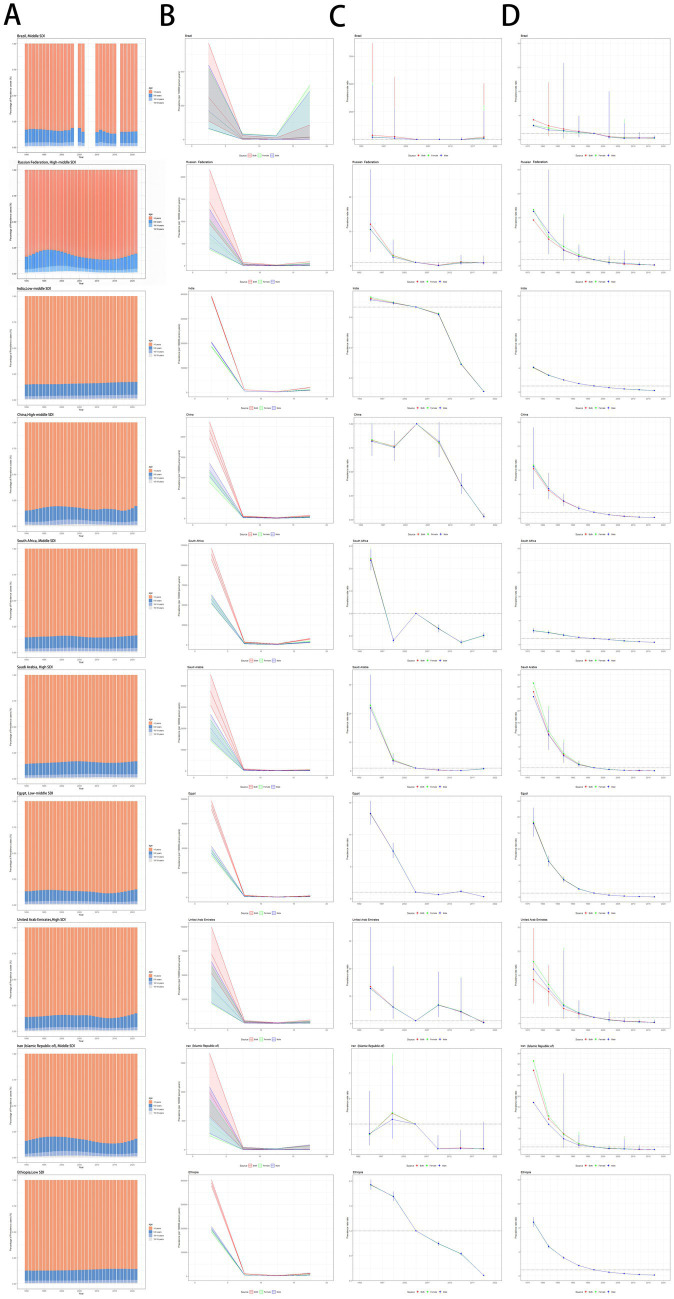
Age distribution of measles prevalence and age-period-cohort effects in the BRICS-plus countries across SDI quintiles. **(A)** Temporal change in the relative proportion of measles across age groups (<5, 5–9, 10–14, 15–19 years), 1990–2021. **(B)** Age effects are shown by the fitted longitudinal age curves of prevalence rate (per 100,000 person-years) adjusted for period deviations. **(C)** Period effects are shown by the relative risk of prevalence rate (prevalence rate ratio) and computed as the ratio of age-specific rates with the referent period set at 2002–2006. **(D)** Cohort effects are shown by the relative risk of prevalence rate and computed as the ratio of age-specific rates with the referent cohort set in 1997. The dots and shaded areas denote prevalence rates or rate ratios and their corresponding 95% CIs. SDI, socio-demographic index; CIs, confidence intervals.

Most measles cases worldwide were recorded in patients under 5 years old, and a similar distribution was observed in almost all BRICS-plus countries (Figure 7A; Supplementary Figures S2A–S4A). From 1990 to 2021, the age distribution of measles cases in the world and BRICS-plus countries was relatively stable, mainly concentrated in people under 5 years old. However, with time, the prevalence, incidence, mortality, and DALYs of measles gradually declined. Among BRICS-plus countries, the number of measles cases in India and Ethiopia was relatively stable, while Russia showed a certain degree of fluctuation.

### 3.6 The effect of age, period, and cohort on prevalence, incidence, mortality, and DALYs

Figures 7B–D; Supplementary Figures S2B–D–S4B–D present the age-period-cohort effect estimates for measles in children and adolescents in BRICS-plus countries. Overall, cohort effects were similar in all countries. After adjusting for period effects, the prevalence and incidence of measles decreased with age in the reference cohort. Almost all countries, except Brazil, showed similar age effects. They leveled off, showing an “L-shaped” curve. In both male and female individuals, the highest prevalence and incidence of measles were found in the 0–5 age group, while BRICS-plus countries, except Brazil, showed lowered prevalence and incidence of measles after 10 years old (Figure 7B; Supplementary Figure S2B). Figure 7C illustrates the estimated cyclical effects by sex over the entire study period. Brazil and Saudi Arabia showed an overall downward trend but a slight rebound between 2017 and 2022. However, the prevalence of measles in Brazil was greatly lower in females than in males, while the opposite pattern was revealed in Saudi Arabia. Russia had the lowest prevalence of measles between 2007 and 2012; India and Ethiopia showed a clear downward trend, with the risk remaining below 1 since 2007; and the risk also remained below 1 in China, except for 2002–2007. The risk remained consistently above 1 in South Africa until 1997 and stabilized at 1 and below since 2002, with a slight upward trend between 2017 and 2022. Egypt consistently had a risk above 1 between 2012 and 2017, showing a slight upward trend. The United Arab Emirates primarily maintained its risk above 1, although it eventually showed a downward trend. Iran had a risk above 1 from 1997 to 2002, trending below 1 since then, and its prevalence remained stable since 2012. Supplementary Figure S2C illustrates the trend in measles incidence among children and adolescents between 1990 and 2021. The incidence in all age groups showed an almost downward trend over time. In particular, the incidence was higher in the 0–5-year age group and lower in the age groups ≥10 years. Figure 7D; Supplementary Figure S2D illustrate cohort-based changes in prevalence and incidence for specific age groups. The prevalence and incidence of measles showed a gradual decrease with age. Supplementary Figures S3B–D, S4B–D illustrate changes in mortality and DALYs.

### 3.7 Prediction of measles burden on children and adolescents in the globe and BRICS-plus countries in 2032

**Figure 8:**
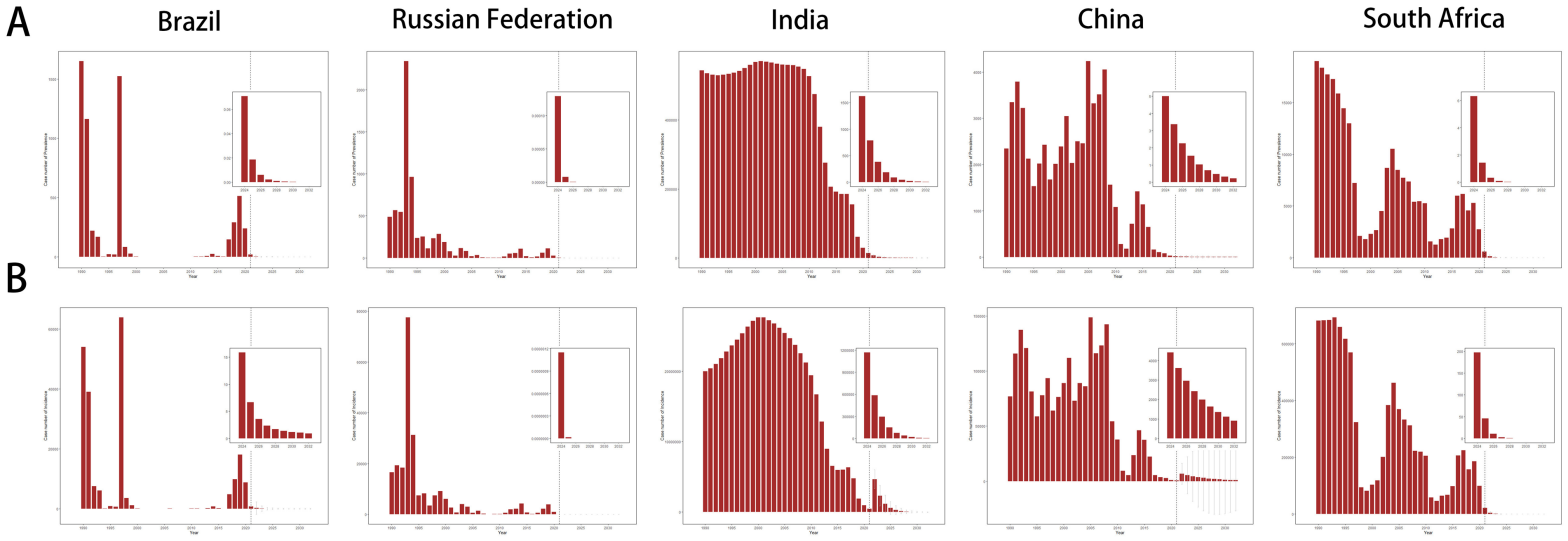
BAPC model prediction of measles burden of children and adolescents in the initial BRICS countries in 2032. **(A)** Forecasted prevalence for measles. **(B)** Forecasted incidence for measles.

BAPC model predictions indicated that during this period, the prevalence and incidence of measles will show a downward trend worldwide. By 2032, the number of overall measles cases will reach 183319.78 (95% UI: −321397.99 to 688037.55) (Supplementary Table 14), and the number of new cases is expected to be 6273361.11 (95% UI: −11029405.35 to 23576127.56) (Supplementary Table 15). Figure 8A shows that the prevalence and number of patients in the initial BRICS countries will continue to decrease. India has the highest prevalence, followed by China, Brazil, and South Africa. In Russia, no one is expected to be infected with measles. Figure 8B shows that the incidence and number of patients in the initial BRICS countries will continue to decrease. The incidence trend is similar in China and India, two countries with large populations, but India shows slightly severe situations. There will be a few new cases in Brazil and South Africa, while Russia shows no new cases. The ARIMA model further confirmed the overall trend in the prevalence and incidence in the five countries mentioned above (Supplementary Figure S5).

## 4 Discussion

Measles is a major cause of death among children in low- and middle-income countries (Stevens et al., 2015; Lopez et al., 2006). This study utilized the GBD database 2021, combined with joinpoint analysis and age-period-cohort models, to reveal the burden of measles on children and adolescents globally and in BRICS-plus countries from 1990 to 2021. Additionally, the BAPC model was employed to predict the trend of measles from 2022 to 2032.

### 4.1 Global and BRICS-plus trends in measles burden

The results showed that the global measles burden among children and adolescents declined significantly during 1990–2021, with the number of measles cases decreasing from 1701026.49 cases in 1990 to 130392.93 cases in 2021, a decrease of 92%, and an average annual decrease of 6.80%. This trend may be related to the increase in global vaccination coverage and public health interventions (Leong and Wilder-Smith, 2019; Auzenbergs et al., 2023). However, there was no significant decline in the prevalence of measles in female patients during 2008–2011, and mortality and DALYs also stagnated, which may be related to the contraction of public health investments in low- and middle-income countries (Andrietta et al., 2020; Addis et al., 2024). The burden of measles in the BRICS-plus countries also showed a significant downward trend. Saudi Arabia showed the most significant decrease in measles prevalence, which reached 100%, with an average annual decrease of 15.20%; in Brazil, the average annual decrease in measles prevalence was 4.09%. Ethiopia showed the highest DALYs (124542.02); the Russian Federation showed the lowest DALYs with only 1.74, indicating that low-income country Ethiopia suffers from a heavy burden of measles, while the high-income country Russia has a very low burden of measles.

Previous studies have confirmed that the risk of measles outbreaks is significantly elevated in areas with less than 95% measles vaccination coverage, leading to a surge in YLLs and YLDs (Fu et al., 2021; Gianfredi et al., 2020). Supplementary immunization targeting specific populations is effective in controlling measles incidence (Kuddus et al., 2023; Sato and Haraguchi, 2021). Between 2000 and 2017, the average annual number of deaths declined by 80% globally due to universal access to MCV (Dabbagh et al., 2018). However, millions of children missed vaccination during the COVID-19 pandemic, resulting in an 18% increase in the number of measles cases and a 43% increase in deaths globally in 2022 compared to 2021, showing signs of an epidemic rebound (Minta et al., 2023). China reported an abnormal surge in measles mortality and DALYs among children and adolescents in 2019–2021, which may be associated with a decline in routine immunization rates during COVID-19, missed vaccination of migrant children, and strained healthcare resources (Wu et al., 2020; Lee et al., 2022; Locke et al., 2023). Recent research findings (Chen et al., 2025) indicate that during the COVID-19 pandemic from 2019 to 2021, the global burden of measles decreased overall, but mortality and DALYs rates in East Asia increased significantly, with EAPC of 155.55 and 146.94, respectively. In addition, the pandemic has disrupted vaccination efforts. Among 204 countries, 75 countries reported a significant decline in the coverage rate for the first dose of MCV, and 68 countries showed a decline in the vaccination rate for the second dose of MCV. We further explored possible influencing factors by conducting a retrospective analysis of the epidemiological situation of measles among children and adolescents in China from 2019 to 2021. This included, but was not limited to, changes in vaccination coverage, population mobility patterns, allocation of medical resources, and socioeconomic factors. By comprehensively analyzing these factors, we hope to gain a more comprehensive understanding of the reasons behind the rise in measles mortality rates and provide data support and recommendations for future public health strategies. We have added the content to the discussion section. The fluctuation in South Africa may be related to the accumulation of susceptible populations, increased population density, and human immunodeficiency virus (HIV) infection, which have led to repeated outbreaks (Sartorius et al., 2013; McMorrow et al., 2009). In addition, political mismanagement and funding deficiency have negatively impacted vaccination (Pustake et al., 2022). Thus, it is evident that the measles burden is influenced by differences in healthcare resources, vaccination coverage, and socioeconomic development.

### 4.2 Negative correlation between SDI and measles burden

SDI is significantly negatively correlated with measles incidence and mortality (Wang et al., 2021; GBD 2015 Maternal Mortality Collaborators, 2016). This study found that the prevalence, incidence, mortality, and DALYs of measles among children and adolescents in the BRICS-plus countries declined significantly as the SDI increased. Among them, Brazil, Russia, China, and Iran had lower-than-expected measles prevalence. High-SDI country Russia (SDI = 0.82) had the lowest prevalence rate (0.01 per 100,000 population), and its vaccination coverage was more than 95%, suggesting that the measles epidemic was effectively controlled (Onishchenko et al., 2011); whereas, Ethiopia (SDI = 0.35) had a low level of economic status and lack of healthcare resources, with vaccination coverage of less than 60%, which resulted in a persistently high burden of measles. This may be closely related to its low vaccination coverage and low level of socio-economic development (Shiferie et al., 2024). Previous studies have noted that rising measles vaccination rates can significantly reduce measles incidence and mortality globally, with the most significant reductions in high-SDI countries, such as Russia and China (Gastañaduy et al., 2021; Wang et al., 2021; Wang H. et al., 2023). Consistently, the present study further confirmed the negative association between SDI and measles burden (George et al., 2024; Bidari and Yang, 2024).

### 4.3 Age and sex patterns analysis of measles in BRICS-plus

The highly contagious nature makes measles a leading cause of childhood deaths globally, especially in areas with low vaccination rates (Lazar et al., 2019; Do et al., 2021; Stein-Zamir et al., 2024). About 350,000 measles cases were reported globally in 2018, resulting in about 142,000 deaths, the majority of which were in children under 5 years of age (Misin et al., 2020). The present study suggested that about 50% of global measles cases occurred in the under-five group, consistent with previous studies (Misin et al., 2020; Wang et al., 2021). This may stem from the fact that infants and young children have immature immune systems and are more susceptible to infection (Simon et al., 2015), indicating the importance of measles vaccination programs for children within 5 years after birth. Ethiopia had the highest prevalence of measles among children under 5 years of age in BRICS-plus countries, followed by South Africa and India. The study also found a significant decline in measles burden with age. DALYs in Ethiopian children under 5 years of age was 820.53 per 100,000 population for males and 630.01 per 100,000 population for females. It declined to 11.88 per 100,000 population and 13.07 per 100,000 population, respectively, in the age group of 10–14 years. This may be attributed to the cumulative effect of the mature immune system and vaccination (Simon et al., 2015; Wang Q. et al., 2023).

### 4.4 Age-period-cohort analysis of measles in BRICS-plus

This study analyzed the prevalence and incidence of measles among children and adolescents in BRICS-plus countries using an age-period-cohort model. The prevalence and incidence of measles in the reference cohort showed a significant age-dependent decline, forming a typical “L-shaped” curve. The 0–5 age group had the highest prevalence and incidence of measles, which was related to the waning of maternal antibodies and the immature immune system (Simon et al., 2015; Wang Q. et al., 2023; Dagan et al., 1995). Although most countries have included the first dose of MCV in the routine immunization program for infants, vaccine interruptions, vaccine hesitancy, or insufficient coverage in some regions may lead to a continued high risk in this age group (Kostandova et al., 2022; Durrheim et al., 2024). Period effect analysis showed that Brazil and Saudi Arabia experienced a rebound in incidence between 2017 and 2022, which may be related to the disrupted routine immunization, vaccine hesitancy, delayed vaccination, and imported cases associated with religious gatherings during the COVID-19 pandemic (Lee et al., 2022; Packham et al., 2024; Chiappini et al., 2021; Shafi et al., 2016). The risk of measles in China, India, and Ethiopia has continued to decline since 2007, indicating the effectiveness of supplementary vaccination campaigns (Auzenbergs et al., 2023; Wang Q. et al., 2023; Shen et al., 2022). Cohort effect analysis further demonstrated that the incidence in more recent birth cohorts was significantly lower than that in earlier cohorts, confirming the cumulative protective effect of vaccination programs (Auzenbergs et al., 2023; Wang Q. et al., 2023). Therefore, precise interventions targeting the 0–5 age group are needed for measles elimination worldwide.

### 4.5 Global and BRICS-plus measles trends by 2032

Predictive models of measles prevalence and incidence globally and in BRICS-plus countries for 2032 revealed the potential progress and persistent challenges in measles elimination. The predictions showed a downward trend in the global number of measles patients, but the wide range of the confidence interval, especially negative values, indicated the sensitivity of the predictive model to public health emergencies. Among BRICS-plus countries, India may maintain the highest prevalence, mainly attributed to the disparity in immunization coverage under its large population base (Panda et al., 2020; Scobie et al., 2015). Although China showed a similar downward trend, densely-populated areas may lead to local outbreaks. The predicted low incidence in Brazil and South Africa is based on the stability of the existing prevention and control systems, but the immunosuppressed state of HIV-infected children in South Africa remains a potential threat (Sartorius et al., 2013; Coetzee et al., 2014). Therefore, future public health policies should continue to increase vaccination coverage, especially in countries with a high measles burden, to further lower the incidence and mortality of measles.

### 4.6 Limitations

Although this study provides a detailed analysis of the measles burden globally and in BRICS-plus countries, there are still certain limitations. Firstly, the GBD database has low data quality in some low-income countries, which may lead to underreporting. Secondly, the timespan (from 1990 to 2021) does not fully consider the impact of the recent decline in vaccination coverage in some countries, especially the rebound in the measles burden from 2019 to 2021, which may be related to interrupted vaccination due to the COVID-19 pandemic. In addition, this study focuses on the relationship between SDI and measles burden but does not deeply explore other influencing factors, such as the allocation of medical resources, population mobility, and changes in public health policies. These factors may have significant impacts on the measles burden in specific countries or regions. Future studies should further explore the independent and interactive effects of these factors to improve the reliability of research results.

## 5 Conclusion

In conclusion, over the past three decades, the burden of measles among children and adolescents in BRICS-plus countries has significantly declined and is closely related to the improvement of SDI. However, there are complex differences in the age, period, and cohort effects of the measles burden among different countries, especially in low- and middle-income countries. For future public health strategies, it is necessary to continuously enhance the vaccination coverage rate, particularly in countries with a heavy burden of measles, such as India and Ethiopia, to further reduce the incidence and mortality of measles. Additionally, all countries need to strengthen measles surveillance systems to ensure the timely detection and control of measles outbreaks and prevent their large-scale recurrence.

## Data Availability

This study provides an in-depth analysis of publicly available datasets. The names and registration numbers of the relevant repositories are as follows: http://ghdx.healthdata.org/gbd-results-tool.

## Footnotes

^1^ https://www.who.int/zh/news-room/fact-sheets/detail/measles

^2^ https://ghdx.healthdata.org/gbd-2021/

## Supplementary material

SUPPLEMENTARY FIGURE S1 Joinpoint regression analyses of measles burden among children and adolescents in Saudi Arabia, Egypt, United Arab Emirates, Iran, and Ethiopia. **(A)** Prevalence cases. **(B)** Incidence cases. **(C)** Mortality cases. **(D)** DALYs cases. DALYs, disability-adjusted life years.

SUPPLEMENTARY FIGURE S2 Age distribution of measles incidence and age-period-cohort effects in the BRICS-plus countries across SDI quintiles. **(A)** Temporal change in the relative proportion of measles across age groups (<5, 5–9, 10–14, 15–19 years), 1990–2021. **(B)** Age effects are shown by the fitted longitudinal age curves of incidence rate (per 100,000 person-years) adjusted for period deviations. **(C)** Period effects are shown by the relative risk of incidence rate (incidence rate ratio) and computed as the ratio of age-specific rates with the referent period set at 2002–2006. **(D)** Cohort effects are shown by the relative risk of incidence rate and computed as the ratio of age-specific rates with the referent cohort set in 1997. The dots and shaded areas denote incidence rates or rate ratios and their corresponding 95% CIs. SDI, socio-demographic index; CIs, confidence intervals.

SUPPLEMENTARY FIGURE S3 Age distribution of measles deaths and age-period-cohort effects in the BRICS-plus countries across SDI quintiles. **(A)** Temporal change in the relative proportion of measles across age groups (<5, 5–9, 10–14, 15–19 years), 1990–2021. **(B)** Age effects are shown by the fitted longitudinal age curves of death rate (per 100,000 person-years) adjusted for period deviations. **(C)** Period effects are shown by the relative risk of death rate (death rate ratio) and computed as the ratio of age-specific rates with the referent period set at 2002–2006. **(D)** Cohort effects are shown by the relative risk of death rate and computed as the ratio of age-specific rates with the referent cohort set in 1997. The dots and shaded areas denote death rates or rate ratios and their corresponding 95% CIs. SDI, socio-demographic index; CIs, confidence intervals.

SUPPLEMENTARY FIGURE S4 Age distribution of measles DALYs and age-period-cohort effects in the BRICS-plus countries across SDI quintiles. **(A)** Temporal change in the relative proportion of measles across age groups (<5, 5–9, 10–14, 15–19 years), 1990–2021. **(B)** Age effects are shown by the fitted longitudinal age curves of DALYs rate (per 100,000 person-years) adjusted for period deviations. **(C)** Period effects are shown by the relative risk of DALYs rate (DALYs rate ratio) and computed as the ratio of age-specific rates with the referent period set at 2002–2006. **(D)** Cohort effects are shown by the relative risk of DALYs rate and computed as the ratio of age-specific rates with the referent cohort set in 1997. The dots and shaded areas denote DALYs rates or rate ratios and their corresponding 95% CIs. SDI, socio-demographic index; CIs, confidence intervals; DALYs, disability-adjusted life years.

SUPPLEMENTARY FIGURE S5 ARIMA model prediction of measles burden trends among children and adolescents in initial BRICS countries from 2022 to 2032. **(A)** Predicted trends of prevalence. **(B)** Predicted trends of incidence.
