# Supplementary figures and images for: Time trend of measles burden on children and adolescents in BRICS-plus countries from 1990 to 2021 and prediction to 2032
Source: Front Microbiol. 2025 Jul 24;16:1612124. doi: 10.3389/fmicb.2025.1612124 (PMC12328342; doi:10.3389/fmicb.2025.1612124)

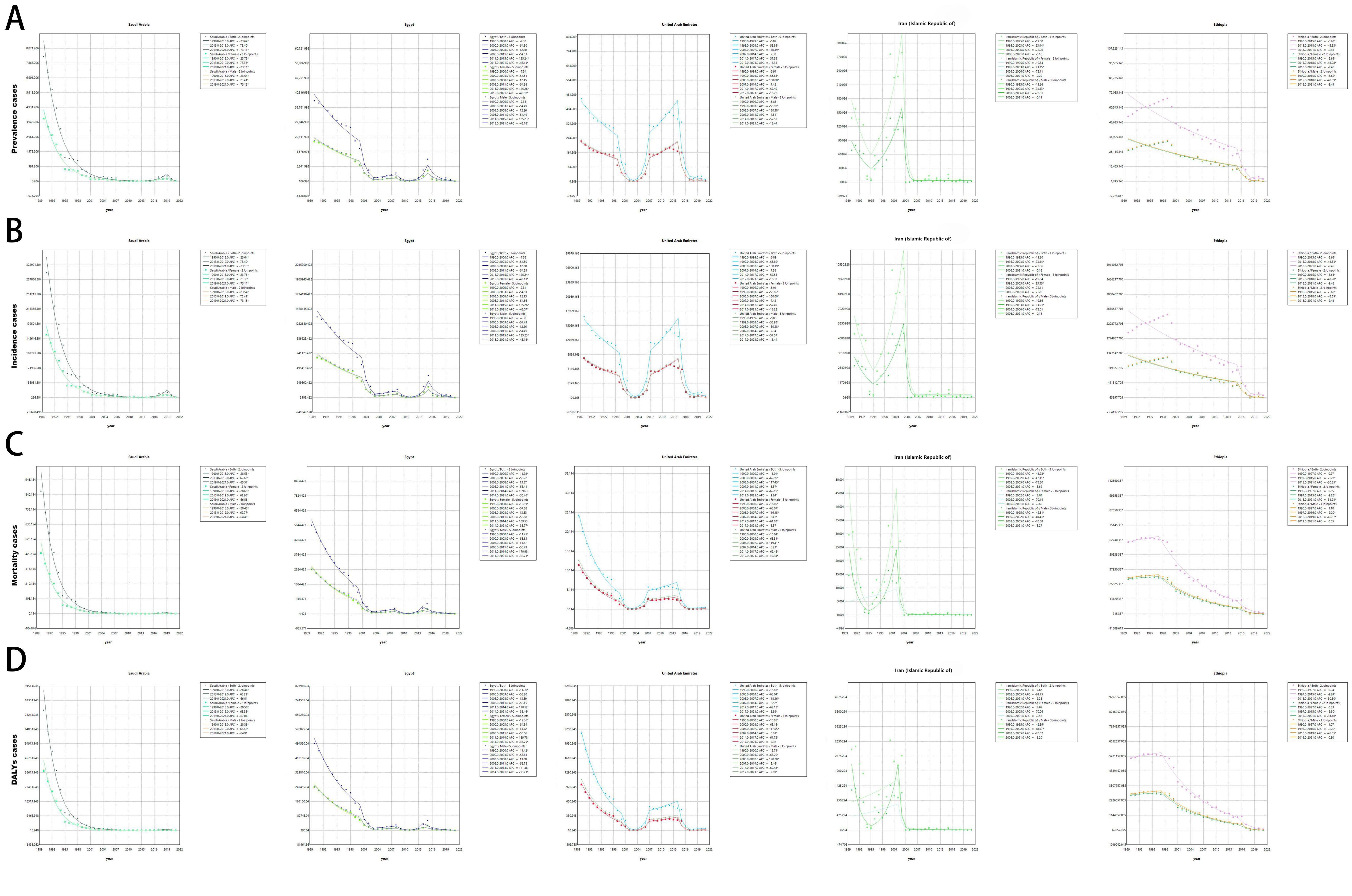

Supplement: SUPPLEMENTARY FIGURE S1 — Joinpoint regression analyses of measles burden among children and adolescents in Saudi Arabia, Egypt, United Arab Emirates, Iran, and Ethiopia. (A) Prevalence cases. (B) Incidence cases. (C) Mortality cases. (D) DALYs cases. DALYs, disability-adjusted life years. [file Image_1.jpg]

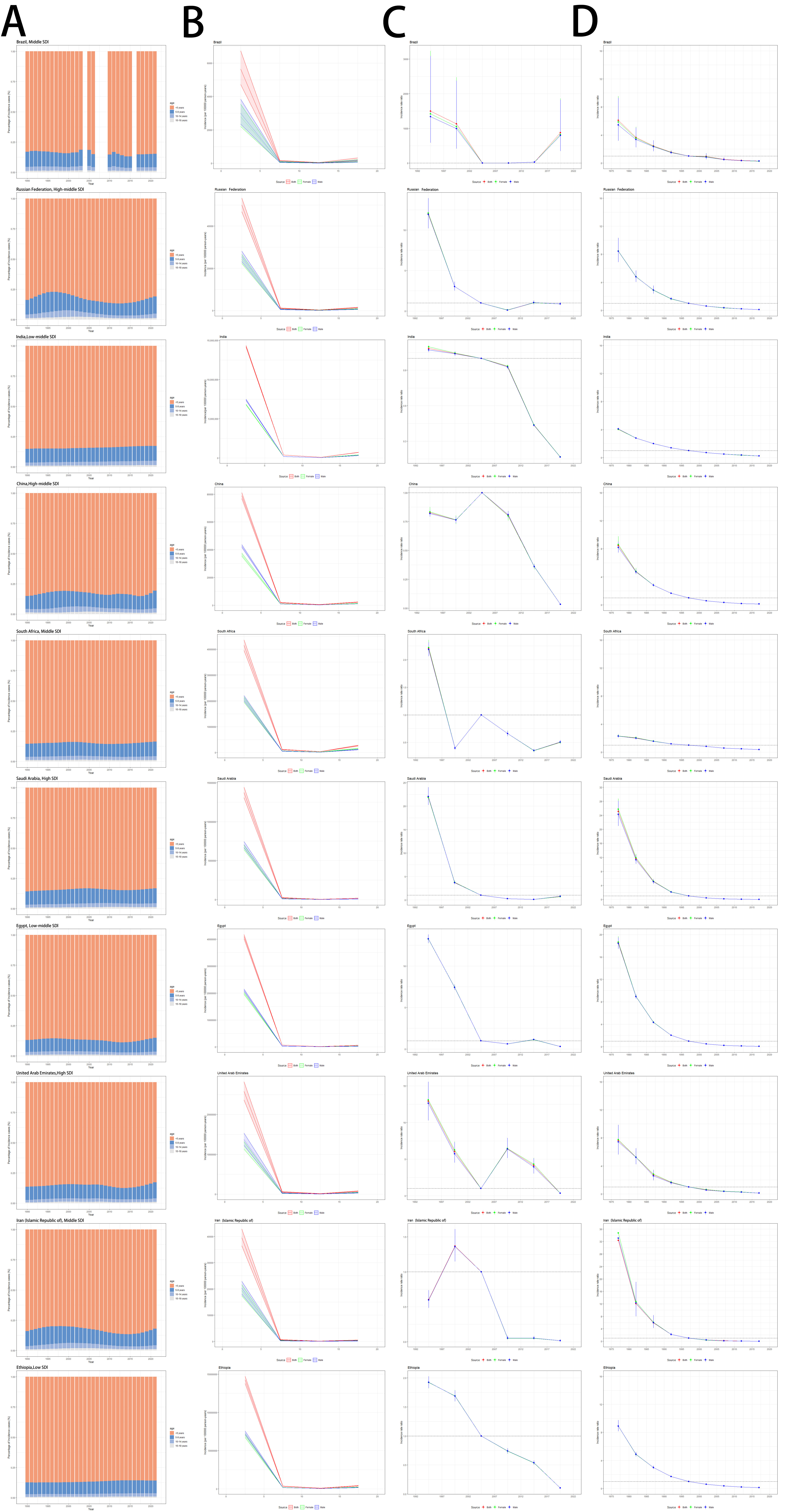

Supplement: SUPPLEMENTARY FIGURE S2 — Age distribution of measles incidence and age-period-cohort effects in the BRICS-plus countries across SDI quintiles. (A) Temporal change in the relative proportion of measles across age groups (<5, 5–9, 10–14, 15–19 years), 1990–2021. (B) Age effects are shown by the fitted longitudinal age curves of incidence rate (per 100,000 person-years) adjusted for period deviations. (C) Period effects are shown by the relative risk of incidence rate (incidence rate ratio) and computed as the ratio of age-specific rates with the referent period set at 2002–2006. (D) Cohort effects are shown by the relative risk of incidence rate and computed as the ratio of age-specific rates with the referent cohort set in 1997. The dots and shaded areas denote incidence rates or rate ratios and their corresponding 95% CIs. SDI, socio-demographic index; CIs, confidence intervals. [file Image_2.jpg]

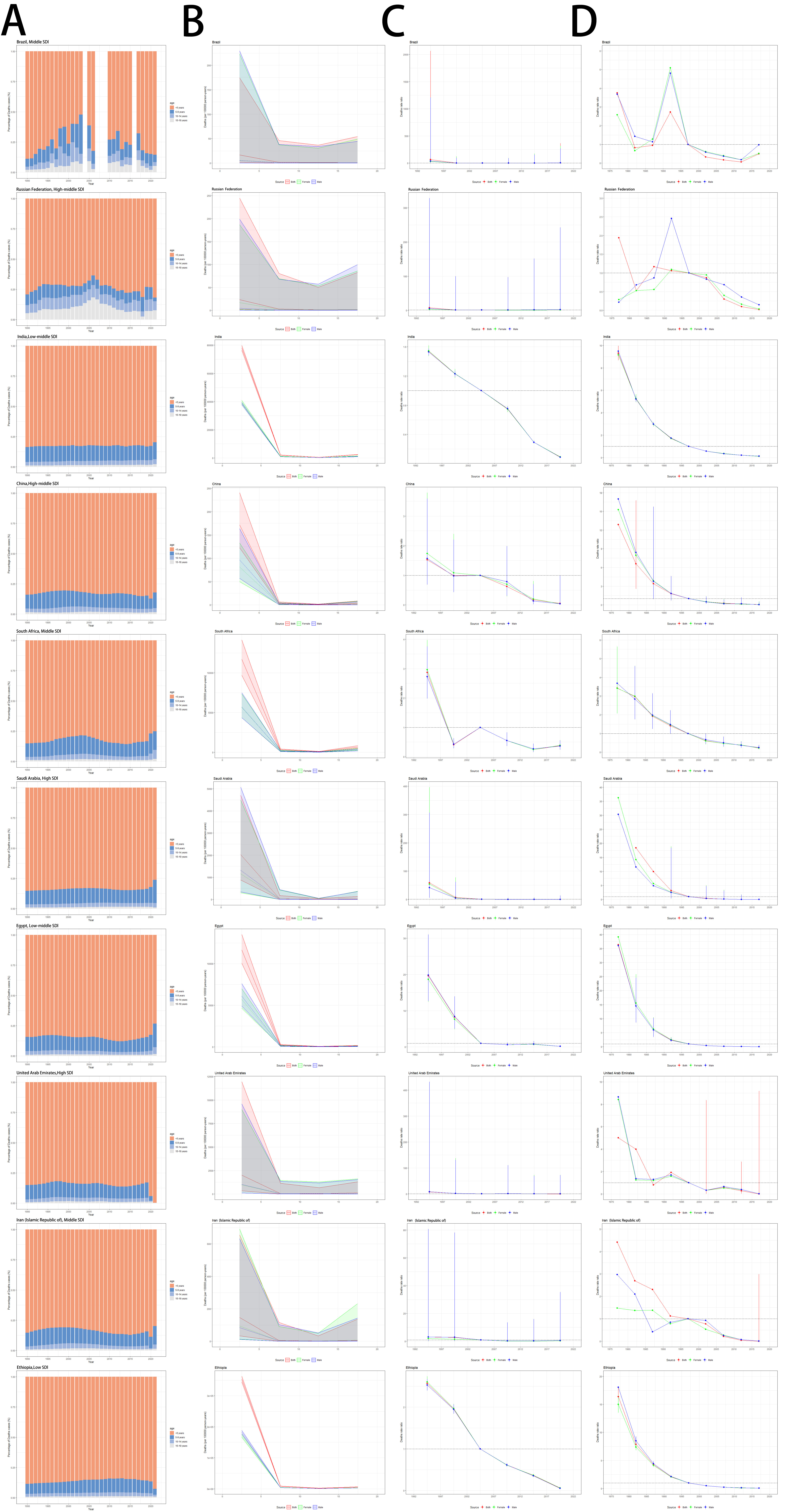

Supplement: SUPPLEMENTARY FIGURE S3 — Age distribution of measles deaths and age-period-cohort effects in the BRICS-plus countries across SDI quintiles. (A) Temporal change in the relative proportion of measles across age groups (<5, 5–9, 10–14, 15–19 years), 1990–2021. (B) Age effects are shown by the fitted longitudinal age curves of death rate (per 100,000 person-years) adjusted for period deviations. (C) Period effects are shown by the relative risk of death rate (death rate ratio) and computed as the ratio of age-specific rates with the referent period set at 2002–2006. (D) Cohort effects are shown by the relative risk of death rate and computed as the ratio of age-specific rates with the referent cohort set in 1997. The dots and shaded areas denote death rates or rate ratios and their corresponding 95% CIs. SDI, socio-demographic index; CIs, confidence intervals. [file Image_3.jpg]

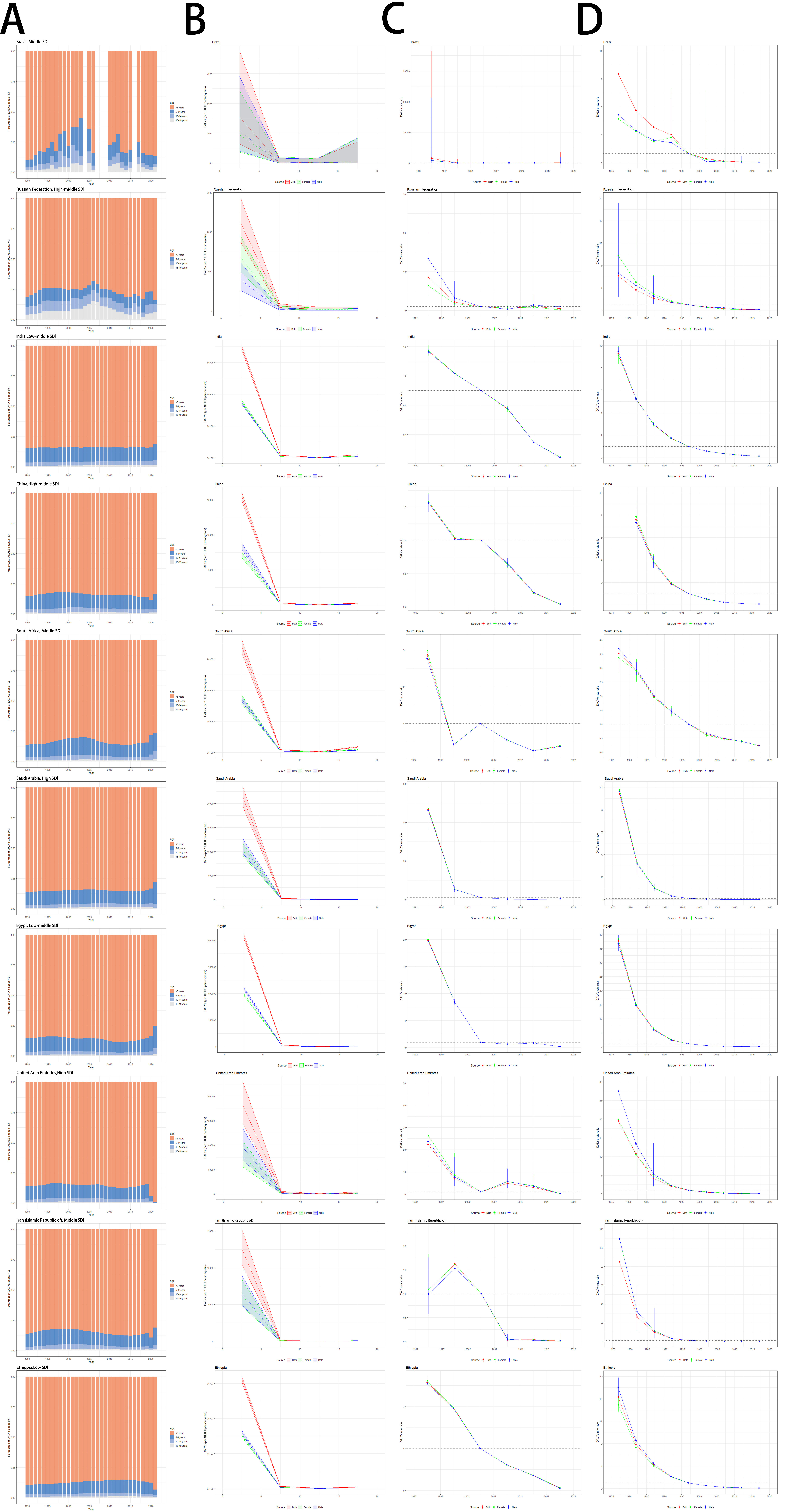

Supplement: SUPPLEMENTARY FIGURE S4 — Age distribution of measles DALYs and age-period-cohort effects in the BRICS-plus countries across SDI quintiles. (A) Temporal change in the relative proportion of measles across age groups (<5, 5–9, 10–14, 15–19 years), 1990–2021. (B) Age effects are shown by the fitted longitudinal age curves of DALYs rate (per 100,000 person-years) adjusted for period deviations. (C) Period effects are shown by the relative risk of DALYs rate (DALYs rate ratio) and computed as the ratio of age-specific rates with the referent period set at 2002–2006. (D) Cohort effects are shown by the relative risk of DALYs rate and computed as the ratio of age-specific rates with the referent cohort set in 1997. The dots and shaded areas denote DALYs rates or rate ratios and their corresponding 95% CIs. SDI, socio-demographic index; CIs, confidence intervals; DALYs, disability-adjusted life years. [file Image_4.jpg]

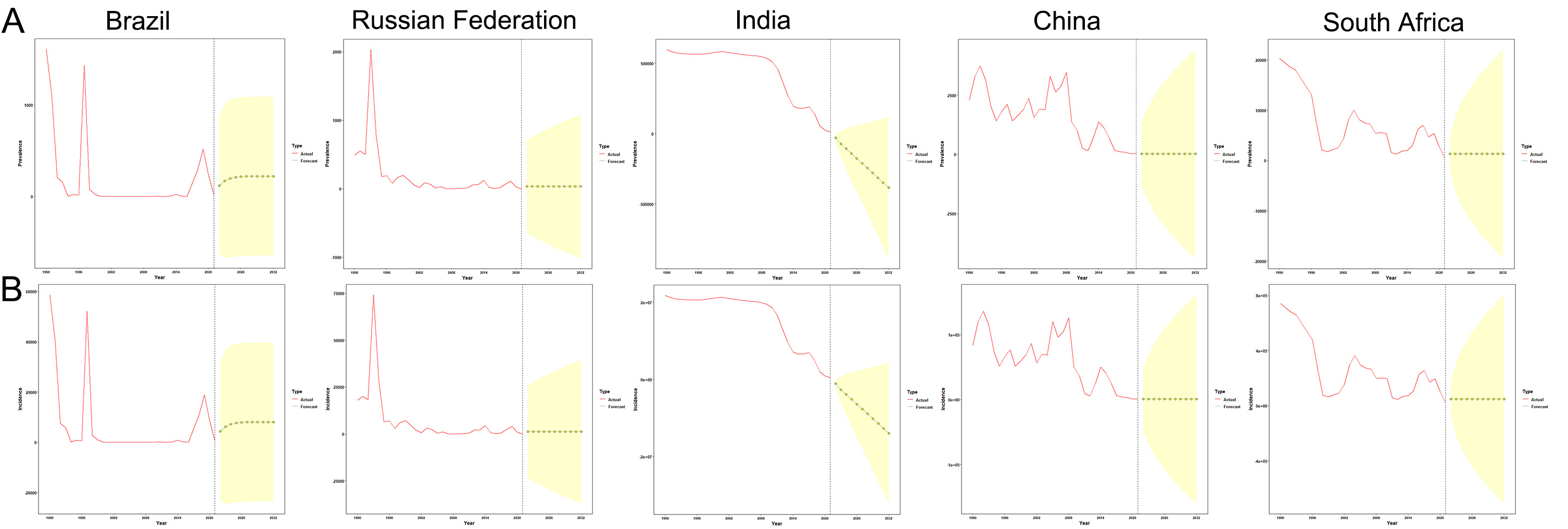

Supplement: SUPPLEMENTARY FIGURE S5 — ARIMA model prediction of measles burden trends among children and adolescents in initial BRICS countries from 2022 to 2032. (A) Predicted trends of prevalence. (B) Predicted trends of incidence. [file Image_5.tif]
